# Cathepsin B launches an apoptotic exit effort upon cell death-associated disruption of lysosomes

**DOI:** 10.1038/cddiscovery.2016.12

**Published:** 2016-02-29

**Authors:** MAG de Castro, G Bunt, FS Wouters

**Affiliations:** 1 Laboratory for Molecular and Cellular Systems, Institute of Neuropathology, University Medical Center Göttingen, Göttingen, Germany; 2 Clinical Optical Microscopy, Institute of Neuropathology, University Medical Center Göttingen, Göttingen, Germany; 3 Centre for Nanoscale Molecular Physiology of the Brain (CNMPB), Göttingen, Germany

## Abstract

The release of cathepsin proteases from disrupted lysosomes results in lethal cellular autodigestion. Lysosomal disruption-related cell death is highly variable, showing both apoptotic and necrotic outcomes. As the substrate spectrum of lysosomal proteases encompasses the apoptosis-regulating proteins of the Bcl-2 family, their degradation could influence the cell death outcome upon lysosomal disruption. We used Förster resonance energy transfer (FRET)-based biosensors to image the real-time degradation of the Bcl-2-family members, Bcl-xl, Bax and Bid, in living cells undergoing lysosomal lysis and identified an early chain of proteolytic events, initiated by the release of cathepsin B, which directs cells toward apoptosis. In this apoptotic exit strategy, cathepsin B’s proteolytic activity results in apoptosis-inducing Bid and removes apoptosis-preventing Bcl-xl. Cathepsin B furthermore appears to degrade a cystein protease that would otherwise have eliminated apoptosis-supporting Bax, indirectly keeping cellular levels of the Bax protein up. The concerted effort of these three early events shifts the balance of cell fate away from necrosis and toward apoptosis.

## Introduction

The loss of lysosomal integrity has been implicated as a decisive step in various forms of cell death that span the width of apoptosis to necrosis.^1–3^ In the first description of lysosomal function, de Duve^4^ already recognized the destructive potential of the lysosomal proteases when he called lysosomes the cell’s 'suicide bags'. Indeed, lysosomal rupture induced by, for example, photodynamic therapy^5–9^ or lysosomophilic detergents^10–13^ is known to cause cell death. Shear force-mediated tearing of lysosomal membranes through the induced rotation of membrane-bound LAMP1 antibody-bound superparamagnetic iron oxide nanoparticles also results in cell death.^14^

The important role of lysosomal rupture in cell death is exemplified by the fact that it appears to be involved in a number of diseases, for example, in neurodegenerative diseases^15^ like Alzheimer’s disease,^16^ and cell death during neuronal ischemia, hypoxia, stroke or trauma.^17–19^ Furthermore, a diminished lysosomal structural integrity has been suggested to underly the increased susceptibility of cancer cells to cell death.^20,21^

The release of lysosomal cathepsin proteases strongly impacts cellular physiology as these proteases are – by their nature – broadly specific. Their main task is the degradation of endocytosed extracellular matrix components and autophagocytosed proteins, as well as intracellular multi-protein structures such as organelles.^22^ The broad degradation of structural and functional protein targets finally leads to necrotic cell death, as characterized by the uncontrolled loss of cellular functions, such as energy production and osmotic balance, and loss of cellular structural integrity. Activation of the caspase family of proteases, on the other hand, engages carefully orchestrated cell death-effector systems in order to achieve the cell's controlled suicide and contained removal from the tissue.^23^ This programmed or apoptotic cell death form can be triggered by receptor-mediated exogenous or cell-stress-related endogenous signals.^24^ In order to serve apoptotic cell death, caspases have acquired a high selectivity for their substrates.^25^ Despite their opposite positions in the cell death spectrum and their widely different operational modes, connections between both proteolytic systems exist. Cathepsins have been implicated in the degradation of apoptosis-regulating proteins of the B-cell lymphoma-2 (Bcl-2) family,^7,26–29^ and targeted lysosomal rupture appears to be able to initiate both apoptotic and non-apoptotic forms of cell death, apparently depending on the extent and/or duration of lysosomal damage.^9,30^ Notably, pathological conditions involving the loss of lysosomal integrity, like ischemic cell death in stroke^31^ or hypoxic regions of solid tumors^32^ possess clear apoptotic and necrotic components. These necroapoptotic connections suggest that some cathepsins exhibit a certain degree of physiological specificity, as supported by specificity-profiling studies.^33,34^ As apoptosis-regulating proteins of the Bcl-2 family have been reported to be digested by cathepsins,^26^ the interplay of both proteolytic systems might have a role in directing the programmatic advancement of the cell death response.

We therefore reasoned that the real-time investigation of the fate of apoptosis-regulating proteins upon lysosomal rupture could provide insights into the specificity of cathepsins, their involvement in programmatic steps and the underlying mechanism that decides between apoptotic and necrotic outcomes. Selective degradation of anti-apoptotic proteins by the cathepsins could support an apoptotic drive under the otherwise necrotic stimulus of lysosomal disruption. To assess these questions, we designed Förster resonance energy transfer (FRET)-based biosensors for the imaging of the degradation of apoptosis-regulating proteins of the Bcl-2 family in living cells.

## Results

### Cleavage of the Bcl-2 proteins Bid, Bax and Bcl-xl upon NDI-induced lysosomal rupture

The proteolytic degradation of BH3 interacting domain death agonist (Bid), Bcl-2-associated X protein (Bax) and Bcl-xl was probed using FRET constructs that consisted of the full protein, sandwiched between N-terminal (donor) mTFP and C-terminal (acceptor) Venus fluorescent proteins. All three biosensors showed a robust cytoplasmic expression and the FRET efficiency in these constructs was 20–30%, as measured by acceptor photobleaching. For our live-cell FRET measurements, we monitored the normalized ratio of sensitized emission fluorescence over the emission of the Venus acceptor upon its direct excitation.^35^ A reduction in the ratio represents cleavage of the constructs.

Lysosomal rupture was chemically induced in MCF-7 cells by the addition of 70 *μ*M N-dodecyl-imidazole (NDI), a lysosomophilic detergent that gains emulsifying action upon its concentration and protonation in the inner lysosomal leaflet.^36^ Lysosomal integrity was analyzed on the basis of the loss of punctate staining with the vital dye Lysotracker Red. At this concentration, lysosomal rupture shows a sigmoidal progression, which is completed around 1 h of incubation with half of the lysosomes being disrupted around 30 min. A swelling response of the cells that is typical for necrotic cell death was seen by phase-contrast imaging from 45 to 60 min. Cell rupture occurs around 90–100 min, with loss of cytosolic content (Supplementary Figure S1).

**Figure 1:**
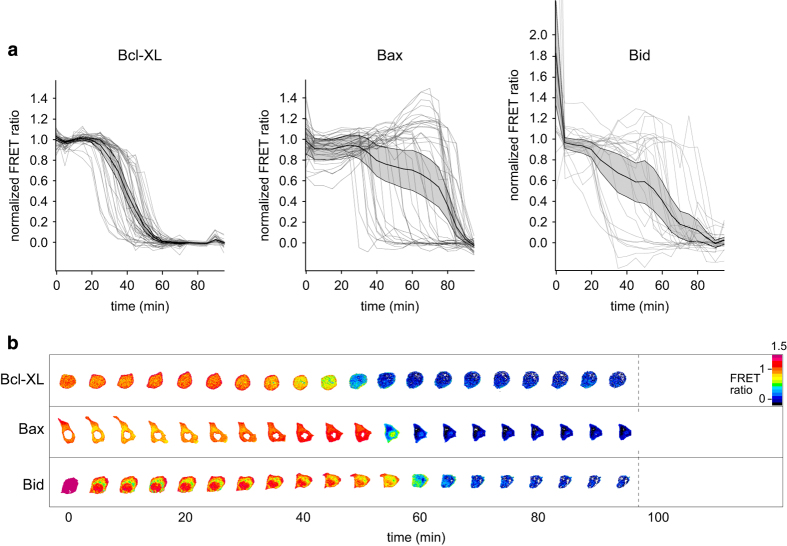
Cleavage of the apoptosis-regulating proteins Bcl-xl, Bax and Bid upon lysosomal lysis. (**a**) Temporal response of the degradation of Bcl-xl (left), Bax (middle) and Bid (right) monitored by FRET in individual cells upon NDI treatment (at time point 0 min). The thick black trace shows the average behavior with the 95% confidence interval shown in the gray area. (**b**) Representative FRET ratio images of the constructs during NDI treatment. Lower ratios (cooler colors) indicate the cleavage of the sensors. Scale bar, 10 *μ*m.

All three biosensor constructs showed inverse sigmoidal FRET time profiles upon addition of NDI (Figure 1). Within each biosensor class, individual cells exhibit variable delays to the onset of cleavage, which then progresses rapidly. Cleavage of the Bcl-xl sensor in the different cells occurs in a relatively narrow time window between ~30 and 60 min after addition of NDI. Bax cleavage behavior is more heterogeneous, with a broadened time window, between ~30 and 90 min. The Bid sensor shows a strikingly different behavior, in which cells show a pronounced and very rapid cleavage in the first minutes upon addition of NDI, followed by a second cleavage response that, on average, is similar to that observed for Bax. For the comparison of the global behaviors of the three biosensors, we plotted the duration of cleavage against the time of maximal activity for each cell. These values are obtained by fitting the cell FRET responses with a Gauss error function (Supplementary Figure S2A), in which these parameters are represented by the standard deviation and average, respectively (Supplementary Figure S2B). This analysis shows that Bax degradation takes place at the latest times but its cleavage lasts shorter than for the other constructs. Bid cleavage (in the second wave) shows the largest variability in duration. Bcl-xl cleavage, though on average starting before the other constructs, is slower in comparison.

The rapid initial cleavage of the Bid construct for the different cells is ~60% (Supplementary Figure S2C), followed by a plateau phase of a second, inverse sigmoidal wave (Figure 1). This difference in timing suggests the involvement of different proteases with activity optima at different times, likely also cleaving Bid at different sites. It should be noted that the two cleavage events are sensed by different sensor molecules as FRET in one sensor molecule is eliminated by a single cleavage event.

Overall, the onset of cleavage for all sensor classes lies within a much longer time scale (10–90 min) than the narrow distribution of their cleavage durations (minutes). This non-linear behavior, with the rapid execution of the cleavage process after a long and variable time lag, suggests the existence of a complex regulatory mechanism underlying the cleavage response.

### Early lysosomal rupture is associated with the activation of the apoptotic caspase cascade

**Figure 2:**
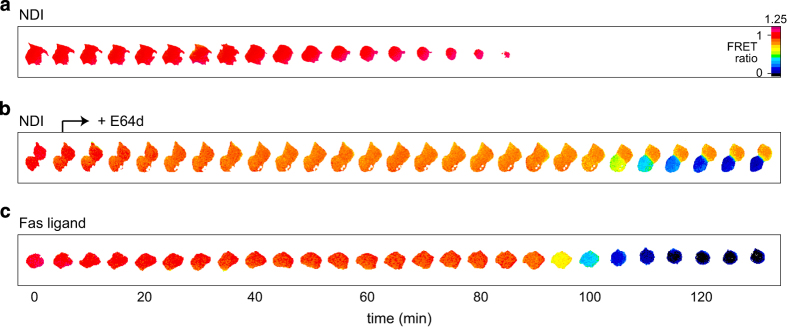
Early, short-lasting cystein cathepsin activation induces apoptosis. The occurrence of apoptosis is monitored by a FRET sensor for active caspase 3/7. (**a**) Treatment with NDI does not induce apoptosis. (**b**) Treatment with NDI, followed by the addition of cystein cathepsin inhibitor E64d after 5 min induces caspase 3/7 activation. (**c**) Apoptosis induced by incubation with trimeric Fas ligand. Lower ratios (cooler colors) indicate sensor cleavage. Scale bar, 10 *μ*m.

In order to evaluate the involvement of apoptotic events upon lysosomal rupture under our experimental condition of 70-*μ*M NDI, we used a caspase 3/7 selective FRET construct. This measurement is based on the proteolysis of the DEVD sequence, sandwiched between cyan and yellow fluorescent proteins. Activation of the executioner caspases 3 and 7 represents the final stages of the apoptotic caspase cascade. Incubation of cells expressing the DEVD construct with NDI did not show a change in FRET ratio during the entire time window of NDI-induced cell death (Figure 2a). Nevertheless, an accumulation of the Bax construct in mitochondria was observed around 30 min of NDI treatment (Supplementary Figure S3), which is generally taken to reflect an early step in the permeabilization of the mitochondrial outer membrane during apoptosis.^37^ These results suggest that at least part of the intrinsic apoptotic pathway had been initiated but that the treatment with NDI, and the lysosomal rupture that it effects, does not mount a successful apoptotic program. As MCF cells express the Fas receptor, as a control, a strong apoptotic response was initiated by the addition of pre-trimerized Fas ligand, leading to the direct activation of the extrinsic apoptotic pathway. This treatment resulted in the cleavage of the DEVD construct as judged from the reduction in its FRET ratio (Figure 2c). The proteolytic profile shows a delay of ~90 min that is followed by rapid degradation, a behavior that has been called 'snap-action variable-delay switching'.^38^ This behavior is similar to our observed degradation of the Bcl-2-family biosensors.

Upon treatment of cells with NDI for 5 min and the subsequent application of the thiol protease inhibitor E64d to stop proteolytic activity of the most abundant thiol cathepsins B, H and L, we were able to detect caspase 3/7 activity in cells (Figure 2b). In this experimental setup, lysosomal proteases were allowed to be active 'only' in the first 5 min of NDI incubation. Remarkably, under these conditions, a rapid caspase cleavage response also started at a time point around 90 min, similar to the direct activation of the extrinsic apoptotic pathway by the Fas ligand (Figure 2c). This supports the hypothesis that an early cathepsin activity peak, reflected by the rapid selective degradation of Bid in the first 5 min, could drive an apoptotic program.

### Pharmacological inhibition of cathepsins uncovers the pro-apoptotic activity of Cathepsin B

**Figure 3:**
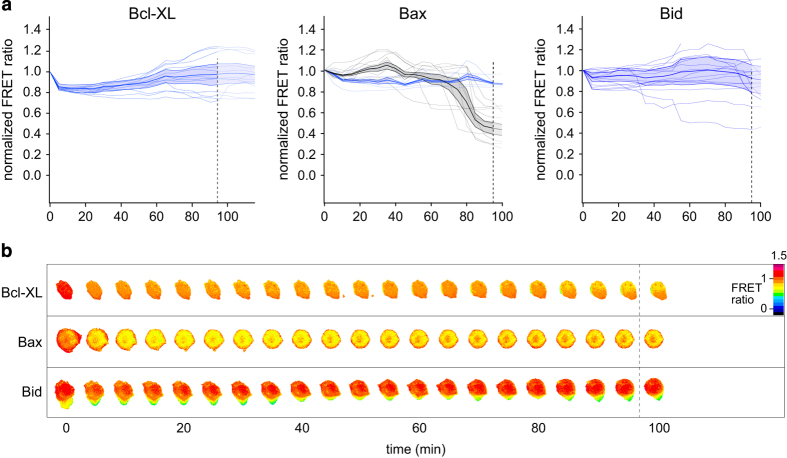
Cleavage of Bcl-xl, Bax and Bid upon lysosomal lysis in the presence of the cystein cathepsin inhibitor E64d. (**a**) Temporal response of the degradation of Bcl-xl (left), Bax (middle) and Bid (right) monitored by FRET in individual cells (gray traces) upon NDI treatment in the presence of the cystein cathepsin inhibitor E64d. The average cellular traces are color coded to indicate different behavioral classes: blue traces do not show cleavage, black traces show delayed cleavage. (**b**) Representative FRET ratio–time series of cells in the dominant, inhibited cleavage (blue trace) class. FRET ratios are shown in false color. Lower ratios (cooler colors) indicate cleavage. Scale bar, 10 *μ*m.

The previous experiments suggest that cells undergoing lysosomal rupture initially attempt to mount an apoptotic response. Nevertheless, in the advancing course of lysosomal disruption to completion, this more favorable response could become overwhelmed by the action of ceaselessly released lysosomal proteases, instead resulting in uncontrolled necrotic cell death. Incubation of the cells expressing the different Bcl-2 family member FRET constructs with E64d either prevented their degradation or expanded the survival time during NDI treatment (Figure 3). The Bcl-xl and Bid sensor show a complete inhibition. In the case of Bid, both the first and the second delayed response were affected. For the Bax construct, the majority of cells (70%) still showed residual degradation, but the onset of their cleavage was delayed and only occurs after about 80 min. The degradation responses observed for Bid, Bax and Bcl-xl are thus likely attributable to lysosomal thiol cathepsins, thereby excluding a significant role of the abundant aspartyl cathepsin D.

As only Bid is significantly degraded in the first minutes after onset of lysosomal rupture, cathepsin activity appears to possess some selectivity. A likely candidate for this early pro-apoptotic response is cathepsin B (CTSB) as it is abundant and neutral active, that is, in a time when most lysosomes are still intact (Supplementary Figure S1).

**Figure 4:**
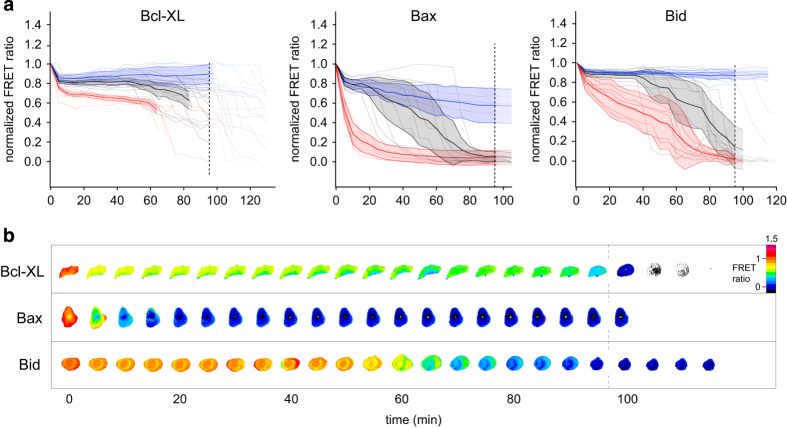
Cleavage of Bcl-xl, Bax and Bid upon lysosomal lysis in the presence of the CTSB-specific inhibitor CA-074Me. (**a**) Temporal degradation response of Bcl-xl (left), Bax (middle) and Bid (right) monitored by FRET in individual cells (gray traces) upon NDI treatment in the presence of the CTSB-specific inhibitor CA-074Me. Color coding of average cellular traces are as in Figure 3 for inhibited (blue) and delay-cleavage (black) behaviors. The new, red traces indicate a population that exhibit cleavage without prior delay. Notably, for Bax, this is the dominant behavior. (**b**) representative FRET ratio-time series of cells in the new, no delay-cleavage (red) class. FRET ratios are shown in false color. Lower ratios (cooler colors) indicate cleavage. Scale bar, 10 *μ*m.

The application of the specific CTSB inhibitor CA074Me during NDI treatment (Figure 4) produced an unexpected outcome. First, the proteolytic profiles of all, Bcl-xl, Bax and Bid, FRET constructs showed three different population behaviors. The first population of cells exhibited a fully inhibited profile similar to treatment with E64d (Bcl-xl: 26%, Bax: 31%, Bid: 38%, Figure 4 blue). A second population of cells still displayed cleavage that was either delayed or comparable to that of NDI without inhibitors (Figure 4 black). The degradation of Bcl-xl was significantly delayed (47%, ~30 min later), for Bid it was less delayed (40%, ~10 min later), and similar in time progression to Bax (16%). Most interestingly, a third population with a new and seemingly paradoxical behavior appeared in which degradation occurred immediately upon NDI addition (Bcl-xl: 27%, Bax: 53%, Bid: 22%, Figure 4 red), without a lag period. Bcl-xl degradation was most inhibited as only small amounts of sensor were degraded in the hour after addition of NDI. As this sensor showed the fastest overall degradation in the absence of protease inhibitors, there is very little overlap between the Ca074Me and non-inhibitor (Figure 1) profiles. For Bid, the specific inhibition of CTSB completely abrogated the prominent degradation peak in the first 5 min, supporting its role in this conversion. Instead, Bid cleavage now proceeded at a monotonously slow speed, with 60% of the construct being degraded at 20–40 min upon NDI addition. For Bax, which underwent the largest behavioral shift upon CA074Me treatment, this new behavior was the predominant population, with very fast cleavage reaching completion at around 20 min, that is*,* earlier than the onset of cleavage in non-treated cells.

This behavior with multiple outcomes for the proteolytic processing of the same substrate is suggestive of a delicate balance in the regulation of the activity of cathepsins. CTSB is inhibited by both inhibitors, CA-074Me and E64d. CA-074Me inhibits specifically, and E64d because it inhibits the thiol cathepsin group of which CTSB is a member. Yet, whereas E64d causes cessation of proteolysis of our sensors, specific inhibition of CTSB is capable of inducing an accelerated proteolytical behavior in many cells across the different Bcl-2-family sensors, most notably for Bax. The role of CTSB in a programmatic cell death pathway thus appears to involve direct and indirect actions. Besides the specific cleavage of Bid, it appears to also cleave another, as yet unidentified, thiol cathepsin. Our experiments suggest that this protease is part of the substrate spectrum of E64d, is highly active and targets Bax. The early responses upon lysosomal lysis that involve CTSB are compatible with a drive toward apoptosis by direct activation of the pro-apoptotic Bid and secondly by preventing the cleavage and removal of Bax.

## Discussion

In recent years, it has become clear that the binary distinction between apoptotic and necrotic cell death only describes the extreme possibilities of a spectrum of cell death forms with apoptotic and necrotic features.^39–41^ A deeper understanding of the nature of the switch between necrotic and apoptotic forms of cell death involving lysosomal rupture is of immediate therapeutic importance as it could help formulate and optimize treatment protocols.

For our FRET-imaging studies, Bid and Bax serve as the major representatives of pro-apoptotic regulators. In response to the induction of apoptosis, Bid binds to Bax to mediate the insertion of Bax into the mitochondrial membrane. Here, Bax forms an oligomeric pore that releases cytochrome c and other pro-apoptotic proteins into the cytoplasm to activate the caspases.^42,43^ Bcl-xl was selected because of its potent function as (anti-apoptotic) survival protein. Bcl-xl is thought to interfere with pro-apoptotic agents like Bid^44^ and Bax^45–47^ to prevent the formation of mitochondrial pores.

**Figure 5:**
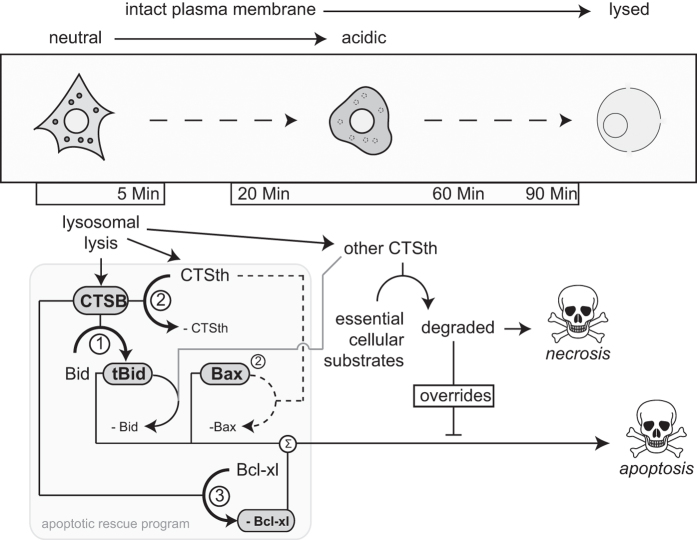
Model of the programmatic regulation of cell death during lysosomal lysis. The observed results with the degradation of the three studied bcl-2-family apoptosis-regulating proteins are summarized in their relation and in time. Numbers refer to the three actions in the rescue program as used in the discussion. CTSth refers to the Bax-degrading thiol cathepsin that is degraded by CTSB.

Our work shows that CTSB mounts an apoptotic 'rescue' program in cells undergoing lysosome-mediated cell death by affecting the apoptosis-regulating Bcl-2 family at three levels in the initial phase of lysis (Figure 5). Concomitant to the proteolytic activation of Bid (tBid), CTSB leads to degradation of the anti-apoptotic protein Bcl-xl. The rescue program also appears to involve a proteolytic chain with another thiol cathepsin as inhibition of CTSB suggests that this co-released cathepsin would have degraded Bax, and to a lesser extent, Bid. CTSB degradation of this thiol cathepsin would thus constitute a disinhibition of apoptosis signaling. The action of CTSB thus appears to involve the production of a pro-apoptotic reactant, its protection against degradation and the removal of anti-apoptotic reactant. This allows the cell to formulate a programmatic response aimed at sustaining a strong apoptotic drive in the background of necrosis-inducing cell damages that involve the loss of lysosomal integrity.

CTSB has been suggested to mimick the apoptosis-supporting selective limited proteolysis of Bid by caspase 8 early during apoptosis, which is known as the 'Bid-shunt'.^48,49^ Cathepsin cleavage sites have been identified in Bid close to the caspase 8 cleavage site that produces the active, truncated form of Bid (tBid).^26^ Furthermore, TNF-α-mediated hepatocyte apoptosis is defective in CTSB knock-out mice.^48^ CTSB thus appears to have a mechanistic role in the induction of apoptosis and lysosomes are likely mediators in the extrinsic pathway of apoptosis.^49,50^ As lysosomal damage is also part of the intrinsic pathway to apoptosis,^51^ the question arises whether CTSB-mediated tBid production can, by itself, sufficiently drive apoptosis in the absence of prior activation of early apoptotic signaling in the extrinsic pathway. Our results show that Bid proteolysis by CTSB can occur independent of prior caspase 8 activation. Besides 'shunting' extrinsic and intrinsic signaling, it could also constitute a starting point for the intrinsic pathway. The degradation of Bid by lysosomal proteases has been shown before under the conditions of lysosomal disruption.^26^ In the latter study, cleavage was shown after 24 h treatment with the LeuLeuOMe detergent, that is, at a late time point when apoptosis was clearly underway. Our work, however, shows extensive cleavage activity immediately after lysosomal permeabilisation, before any apoptotic or necrotic effect can be observed. This finding is in line with an initiating role comparable to caspase-produced tBid.

The progression of proteolytic autodigestion of the cell in the case of lysosomal rupture is likely governed by the acidification of the cytoplasm that accompanies the release of cathepsins. As different cathepsins exhibit different pH enzymatic optima and stabilities,^52^ an inherent sequence in their participation in the progressive digestion of the cell is to be expected. As a considerable degree of permeabilisation is required before the content of the lysosomes is spilled into the large cytoplasmic volume sufficiently for a significant drop in pH to occur, it is obvious that the early phase in NDI treatment must be governed by pH-neutral active cathepsins, such as CTSB, and that the last phase of the cell death pathway can also involve cathepsins with a more acidic enzyme optimum. Our earlier study using the pHlameleon pH sensor has shown that NDI-mediated lysosomal lysis will eventually lower the cytoplasmic pH to 6.5, and in some cells to values as low as 5.5.^53^ In this sense, it is likely that the abundant aspartic cathepsin D, which was not inhibited by E64d, is involved in late proteolytic responses. In fact, some late proteolytic processing of Bax and also Bid was observed when thiol cathepsins were inhibited. Such an inherent sequence might be further supported by timing effects caused by inter-cathepsin degradation as we suggest here for CTSB and a Bax-specific thiol cathepsin, and by the presence of natural cellular inhibitors like the cystatin and stefin family that can buffer a certain amount of active cathepsin released into the cytoplasm.^52^ A study combining experimental perturbation, data modeling and simulation of TNF/TRAIL apoptosis signaling suggests that the variable delay in the progression of apoptosis ('snap-action') stems from cell-to-cell variation in the concentration of apoptotic pathway reactants.^38^ In small volumes like cells, reactions are governed by small numbers of proteins and organelles. Cell-to-cell variation in their composition, for instance due to differences in expression and 'partitioning errors' during cell division can therefore significantly influence the outcome and dynamics of reactions to give rise to population variation.^54,55^ These effects can explain the observed variability in the onset of the degradation of our FRET biosensors. In this light, it should be noted that, even though the onset of the degradation varies, the rate at which the tested Bcl-2-family proteins are then degraded is very similar between cells and show 'snap-action' behavior (Supplementary Figure S2B). Cellular heterogeneity has been suggested to render important biological responses less vulnerable to environmental variation in single decisive parameters.^38,56^

The nature of the Bax-reactive thiol cathepsin that is degraded by CSTB upon their release into the cytoplasm remains unknown, and is the topic of further investigation.

Our work goes toward explaining the observed relationship between the severity of lysosomal damage and the choice between apoptosis or necrosis.^30^ In order to understand the mechanistic basis of an 'emergency brake' on necrosis, it was necessary to observe the proteolytic fate of major players in the Bcl-2 protein family, and to be able to quantitatively record their relative degradation trajectories in the cells as they undergo lysosomolytic death. The variable response in the degradation of these proteins, especially after CTSB inhibition shows the importance of single cell recordings.

Our work contributes to the growing understanding of the role of lysosomes in essential physiological responses to environmental changes. Besides important roles in the removal of function-impaired organelles and potentially dangerous protein aggregates by autophagy, a source for proteases that help shape the extracellular matrix, and a role in antigen processing for MHC-II loading, these organelles and their content have emerging roles in the regulation of apoptosis in a number of cellular settings and treatments. We show that this function is delicately organized such as to optimize the chance for successful apoptotic escape in the case of massive cellular insults. The thiol cathepsin cycle that is initiated by CTSB thus constitutes a new subset of the apoptotic signaling machinery, located at the border between apoptosis and necrosis, that expands the palette of programmatic steps in programmed cell death forms.

## Materials and Methods

### Expression vectors

The Bid cDNA (IRATp970C1135D) was acquired from Imagenes (Berlin, Germany). Bax (pcDNA:HABax) and Bcl-Xl (AAV-6P1-TB) cDNAs were kindly provided by Dr. Lawrence Banks, ICGEB, Trieste, Italy and Dr. Sebastian Kügler, University Medicine Göttingen, Germany, respectively. The full-length cDNAs were transferred to pcDNA3.1, where they were inserted between the coding sequences of mTFP (at N-terminus) and Venus (C-terminus) to produce the proteolytic FRET sensors. The connecting linker sequences between mTFP and the (start codon of the) substrate protein was 5′- gga-3′, encoding for an additional glycin residue, and 5′- gggagt-3′, encoding for the amino acids glycine and alanine, between the last codon of the substrate protein to Venus.

### Culture treatment, transfection and lysosomal staining

MCF-7 cells (human breast adenocarcinoma cell line) were kindly provided by Professor Tony Ng, King's College London, England. MCF-7 cells (100 000 cells/well) were cultured in DMEM medium supplemented with 4.5 g/l glucose, 2 mM L-glutamine, 1 mM pyruvate, 5% FCS and 1% penicillin/streptomycin (all from Gibco, Invitrogen GmbH, Karlsruhe, Germany). For imaging, the MCF-7 cells were grown in Lab-Tek chambered borosilicate cover glass slides (Nalge Nunc International, Penfield, NY, USA), incubated in a humidified atmosphere at 37 °C and 5% CO_2_ to 60–80% confluence and transfected using the Lipofectamine LTX Reagent (Invitrogen GmbH) according to the instructions provided by the manufacturer. For the imaging of lysosomes, cells were stained with the lysosomotropic fluorochrome Lysotracker Red (Invitrogen) at a concentration of 50 nM for 1 h in a humidified atmosphere at 37 °C and 5% CO_2_. Cells were subsequently washed with phosphate-buffered saline and placed in imaging medium (DMEM medium without phenol red, supplemented with 4.5 g/l glucose, 2 mM L-glutamine, 1 mM pyruvate, HEPES pH 7.4 and 1% penicillin/streptomycin (Gibco). Lysosomal rupture was induced by incubation of the cells with 70 *μ*M lysosomotropic detergent N-dodecyl (C12)-imidazole (NDI, Toronto Research Chemicals, Toronto, ON, Canada) in imaging medium for the indicated time periods.

For the inhibition of cathepsin activity, cells were treated with the broad-spectrum cystein protease inhibitor E64d or the specific cathepsin B inhibitor CA-074Me (both from Enzo Life Sciences, Farmingdale, NY, USA), both at a final concentration of 50 *μ*M. For these experiments, transfected cells were incubated for at least 18 h in the presence of E64d or CaO74Me before imaging and these concentrations were maintained throughout the imaging session.

The extrinsic apoptosis pathway was induced by incubation with 120 ng/ml trimeric Fas ligand according to the manufacturer’s instructions (IBA GmbH, Göttingen, Germany).

### Imaging conditions

Live MCF-7 cells were placed in imaging medium (high-glucose DMEM without phenol red supplemented with HEPES pH 7.4 (Gibco)) and imaged at 5 min intervals at 37 °C. Imaging was performed on the Cell Observer Z.1 (Carl Zeiss Microscopy GmbH, Göttingen, Germany) platform equipped with an ECPlan-Neofluor 40X/NA1.3 oil-immersion objective and an AxioCam (MRm) camera (Carl Zeiss Microscopy GmbH). mTFP and Venus were excited by 455 and 505 nm LED illumination, respectively, using the Zeiss Colibri illumination unit. Fluorescence emissions of mTFP and Venus were detected with the bandfilters 494/20 and 542/27. Lysotracker Red was excited with a Zeiss 120 W HXP lamp (Carl Zeiss Microscopy GmbH), using a BP 546/12 filter (AHF Analysentechnik AG, Tübingen, Germany) and emission was detected with a LP 590 filter (AHF Analysentechnik AG).

### Data analysis—FRET

Image processing and ratiometric calculations were performed using a custom-written Python script. Ratiometric FRET was determined by the ratio of sensitized emission over direct acceptor-excitation emission.^35^ Prior to ratio calculations, the background signal was subtracted and the images were thresholded and smoothed with a Gaussian filter (sigma=1). The resulting ratio images were analyzed using ImageJ (http://rsb.info.nih.gov/ij/). Analysis, fitting and data representation were performed using the IgorPro suite (Wavemetrics, Lake Oswego, OR, USA). For comparison of the proteolytic progression between cells and samples, ratio value traces were normalized to their stable start and end values, such that proteolysis progresses from a value of 1 to 0.

The 95% confidence intervals for mean proteolysis profiles of cells expressing one construct and subjected to the same treatment were plotted as (average±1.96 * S.E.M.).

For visualization purposes, the FRET ratios against time (t) were also fitted with the error function

$$FRET\left(t\right)=1-\frac{1}{2}\left(1+erf\left(\frac{\left(t-µ\right)}{\sqrt{\left(2\sigma^{2}\right)}}\right)\right)$$

which transforms the normalized proteolytic profile into a normal distribution of proteolysis rate, from which the time of maximal velocity (its average *μ*) and the duration of the cleavage reaction (its standard deviation *σ*) is obtained.

### Data analysis—lysosomal integrity

Lysosomal integrity was judged from the Lysotracker Red staining of cells during the time course of NDI treatment. As lysosomal leakage progresses, the fluorescence signal changes from a clear punctate to a homogeneous distribution. This progression is described by a reduction in the standard deviation of the average staining signal in the images (determined using ImageJ). Intensities were normalized to the initial signal intensity.

## Abbreviations

Bcl-2: B-cell lymphoma-2
Bax: Bcl-2-associated X protein
Bid: BH3 interacting domain death agonist
cDNA: complementary deoxyribonucleic acid
CTSB: cathepsin B
DEVD: asparagine-glutamine-valine-asparagine
DMEM: Dulbecco’s modified Eagle medium
FCS: fetal calf serum
FRET: Förster resonance energy transfer
HEPES: 4-(2-hydroxyethyl)-1-piperazineethanesulfonic acid
LAMP1: lysosomal-associated membrane protein 1
MCF-7: Michigan Cancer Foundation-7
mTFP: monomeric teal fluorescent protein
NDI: N-dodecyl-imidazole.
