# Supplementary material for: Cathepsin B launches an apoptotic exit effort upon cell death-associated disruption of lysosomes
Source: Cell Death Discov. 2016 Feb 29;2:16012–. doi: 10.1038/cddiscovery.2016.12 (PMC4979493; doi:10.1038/cddiscovery.2016.12)
Supplement: Supplementary Information [file cddiscovery201612-s1.doc]

**Supplementary Figure legends**

**Supplementary Figure 1. NDI-induced lysosomal lysis.** (A) Phase contrast images (upper panel) and lysotracer-red staining (lower panel) are shown for cells that have been treated with NDI (A). Note the rounding of cells and the loss of punctate Lysotracer-red staining, to be replaced by a homogeneous cytoplasmic staining. This indicates the progressive loss of the dye from the lysosomes due to their lysis. (B) The time profile of lysis can be monitored quantitatively on the basis of the homogeneity of the fluorescent stain. Bar=10 μm.

**Supplementary Figure 2. Population distribution of degradation responses with NDI.** (A) The temporal traces of cells expressing the different FRET sensors that represent the *amount* of intact sensor over time, are transformed using an error function into Gaussian distributions (right image) that reflect the degradation *activity* over time. From these distributions, the average time of maximal degradation activity (μ), represented by the peak position, and the duration of cleavage, represented by the width of the distribution at half-maximum (σ) are obtained. (B) These two parameters are plotted in their relation to visualize the population behavior of cells expressing the different sensors. The lines connect the values of the individual cells expressing one sensor construct to the population median. This representation visualizes the collective behavior of the different sensor-expressing cells and shows that these differ from each other.

(C) The pronounced drop in FRET ratio for Bid-sensor-carrying cells in the first 5 minutes of NDI treatment is shown in relation to the time of maximal cleavage activity. Most cells show a drop of a factor between 1 and 2, but the magnitude of this drop does not appear correlated with the later maximal time of degradation (μ).

**Supplementary Figure 3. Bax incorporation into mitochondria upon lysosomal lysis.** NDI treatment leads to the translocation of Bax to the mitochondria. This is generally considered to reflect an early step that lead to the loss of mitochondrial outer membrane potential in the apoptotic pathway. This event is not followed through by activation of the executioner caspase 3/7 (Fig 2A), unless cathepsins are inhibited after the first 5 minutes of their activity (Fig 2B). Shown is the CFP fluorescence signal of the Bax FRET construct, using an inverted gray scale lookup table. Bar=10 μm.
