# Supplementary figures and images for: Cathepsin B launches an apoptotic exit effort upon cell death-associated disruption of lysosomes
Source: Cell Death Discov. 2016 Feb 29;2:16012–. doi: 10.1038/cddiscovery.2016.12 (PMC4979493; doi:10.1038/cddiscovery.2016.12)

A

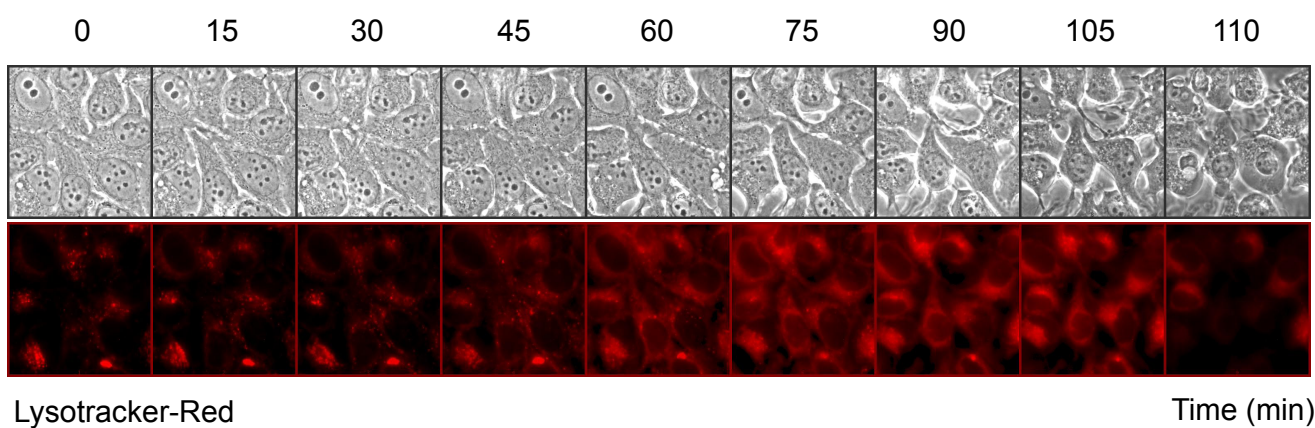

B

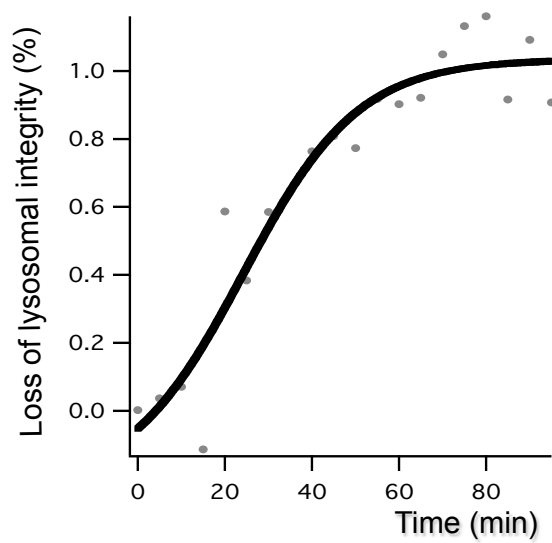

Supplement: Supplementary Figure 1 [file cddiscovery201612-s2.pdf]

A

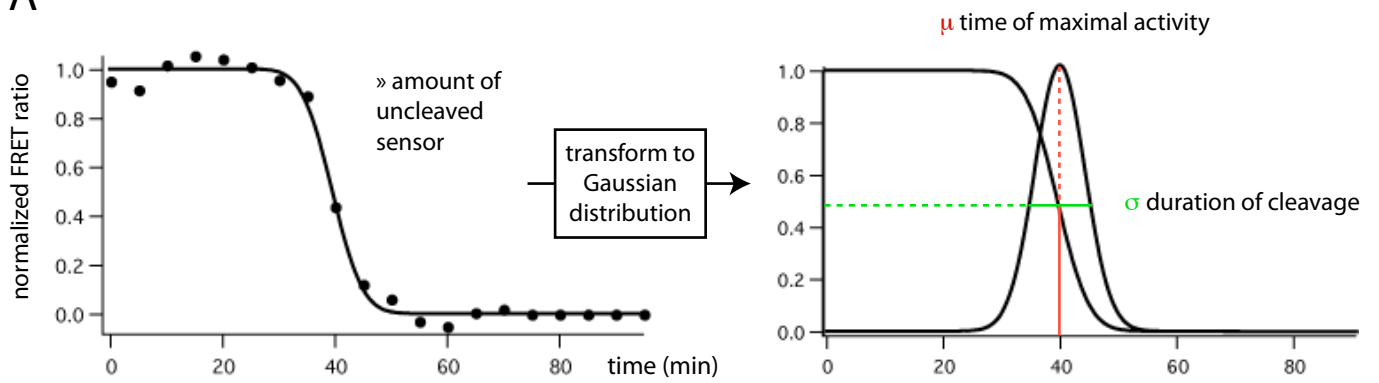

B

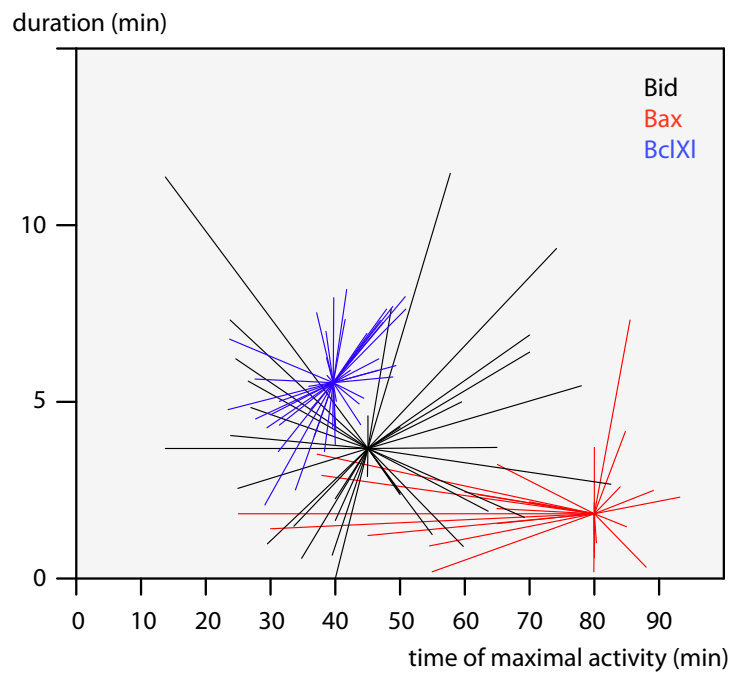

C

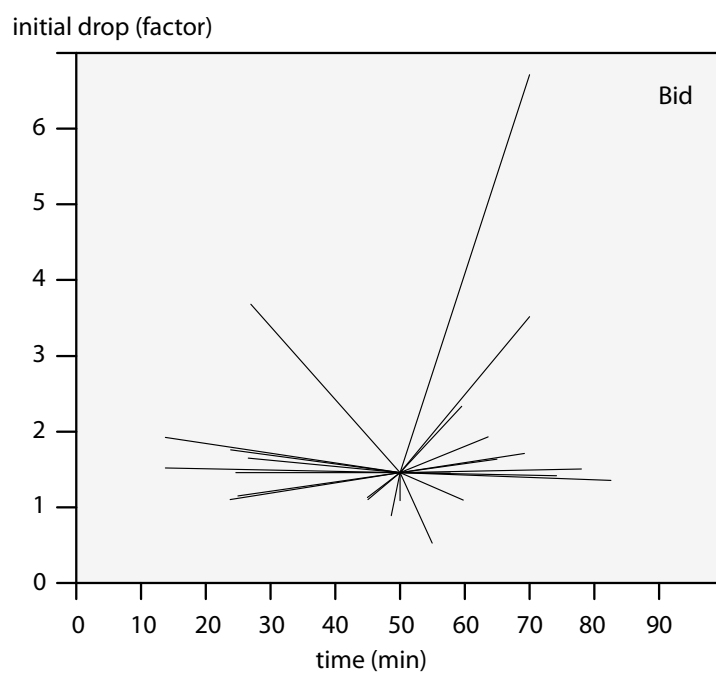

Supplement: Supplementary Figure 2 [file cddiscovery201612-s3.pdf]

0

10

20

30

40

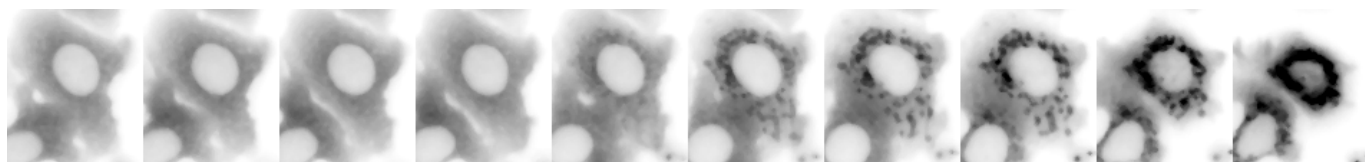

50

60

70

80

90

time (min)

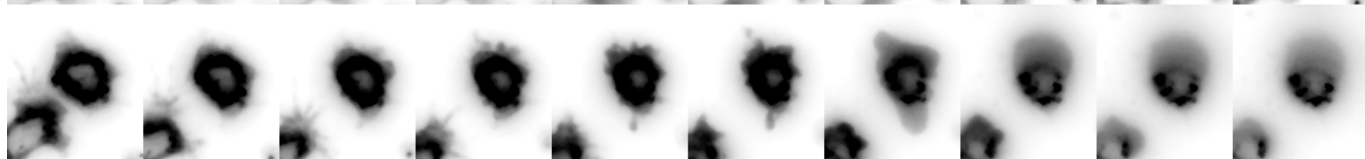

Supplement: Supplementary Figure 3 [file cddiscovery201612-s4.pdf]
